# The Antiviral Effect of Baicalin on Enterovirus 71 *In Vitro*

**DOI:** 10.3390/v7082841

**Published:** 2015-08-19

**Authors:** Xiang Li, Yuanyuan Liu, Tingting Wu, Yue Jin, Jianpin Cheng, Changbiao Wan, Weihe Qian, Fei Xing, Weifeng Shi

**Affiliations:** 1 Department of Clinical Laboratory, Huai’an Hospital Affiliated of Xuzhou Medical College, 62 Huaihai south road, Huai’an, Jiangsu 223300, China; E-Mails: lixiang_suda@163.com (X.L.); huaianjinyue1999@163.com (Y.J.); chengjianping2011@aliyun.com (J.C.); huaianwancb@163.com (C.W.); huaianqianwh@163.com (W.Q.); xingfei115@163.com (F.X.); 2 Department of Endocrinology, Huai’an First Affliated Hospital of Nanjing Medical University, 6 Beijing west road, Huai’an, Jiangsu 223300, China; E-Mail: liuyy_suda@163.com; 3 Department of Clinical Laboratory, the Fourth People’s Hospital of Huai’an, 128 Yanan east road, Huai’an, Jiangsu 223300, China; E-Mail: wutt8870@163.com; 4 Department of Clinical Laboratory, the Third Affiliated Hospital of Soochow University, 185 Juqian street, Changzhou, Jiangsu 213003, China

**Keywords:** baicalin, enterovirus 71, 3D polymerase, apoptosis

## Abstract

Baicalin is a flavonoid compound extracted from *Scutellaria* roots that has been reported to possess antibacterial, anti-inflammatory, and antiviral activities. However, the antiviral effect of baicalin on enterovirus 71 (EV71) is still unknown. In this study, we found that baicalin showed inhibitory activity on EV71 infection and was independent of direct virucidal or prophylactic effect and inhibitory viral absorption. The expressions of EV71/3D mRNA and polymerase were significantly blocked by baicalin treatment at early stages of EV71 infection. In addition, baicalin could decrease the expressions of FasL and caspase-3, as well as inhibit the apoptosis of EV71-infected human embryonal rhabdomyosarcoma (RD) cells. Altogether, these results indicate that baicalin exhibits potent antiviral effect on EV71 infection, probably through inhibiting EV71/3D polymerase expression and Fas/FasL signaling pathways.

## 1. Introduction

Hand, foot, and mouth disease (HFMD) is a common disease in children under five years old, while EV71 is one of the causative pathogens. EV71 is a single positive-strand RNA virus belonging to the enterovirus genus in the Picornaviridae family [1]. EV71 infection occurs throughout the year but is most common in summer and autumn [2,3]. The infection often leads to many neurological diseases, such as aseptic meningitis, brain stem encephalitis, poliomyelitis-like paralysis, and death. To date, there is no effective treatment and there are high rates of disability and mortality due to the lack of effective preventive and treatment drugs [4,5].

Viral infection usually causes host cell damage and apoptosis [6], a highly regulated form of cell death characterized by cell shrinkage, fragmentation, and disposal without loss of plasma membrane integrity and inflammation. The process may be triggered by the interactions of the pro-apoptotic stimuli with various sensors, such as death receptors or the mitochondrial or endoplasmic reticulum pathway [7,8]. As previously reported, coxsackievirus A16, herpes simplex virus 2, and highly virulent H5N1 can induce the apoptosis of RD cells, endothelial cells, and T lymphocytes [9,10,11]. However, little is known about the mechanisms between EV71 infection and RD cell apoptosis. Traditional Chinese medicine shows superiority in fighting viral infection due to its versatile anti-viral activity, for example, directly inhibiting or inactivating viruses, or improving the host immune system [4,12,13]. Baicalin (5,6-dihydroxy-7-*O*-glucuronide flavonoid glycosides) is extracted from *Scutellaria* roots characterized by antibacterial, diuretic, anti-inflammatory, and antispasmodic function [14]. Previous studies showed that baicalin has strong inhibitory activities on dengue virus, hepatitis B virus, influenza virus, and human immunodeficiency virus, but the antiviral effect of baicalin on EV71 infection remains unclear [15,16,17,18].

Here, we found that baicalin could effectively inhibit EV71 replication and protect RD cells from EV71 infection. Moreover, baicalin exhibited a potent antiviral effect on EV71 infection by interfering with 3D polymerase transcription and translation during the early stages of EV71 replication. In addition, baicalin might inhibit EV71-induced apoptosis through regulating the Fas/FasL signaling pathways.

## 2. Materials and Methods

### 2.1. Virus and Chemicals

RD cells were maintained in Dulbecco’s modified Eagle’s medium (DMEM) supplemented with 10% fetal bovine serum (FBS) (Hyclone, Los Angeles, CA, USA) at 37 °C in the presence of 5% CO_2_. When the cells reached 80% to 90% confluency and were in good condition, they were infected with 100 µL of EV71 virus solution (strain BrCr-TR) in 1 mL of serum-free basal medium for 1 h at 37 °C. The cells were washed with PBS three times and cultured in DMEM with 2% FBS. More than 70% of RD cells showed lesions and were then lyzed by freezing and thawing them three times. The cellular debris was removed by centrifuging at 5000 *g* for 10 min at 4 °C. The virus titers were measured by plaque reduction assay. Baicalin was purchased from Aladdin Company (Shanghai, China) with a purity level of ≥98% (HPLC), dissolved in DMSO, and stored at −20 °C for further experiments.

### 2.2. Antibodies

EV71/2A and 3C mouse polyclonal antibodies were kindly provided by Dr. L. Zhang and Dr. J. Liu (Institute of Laboratory Animal Sciences, Chinese Academy of Medical Sciences) [19]. EV71/VP1 and 3D rabbit polyclonal antibody were purchased from Biosynthesis Company (Beijing, China). FasL, caspase-3, and NF-κB p65 were provided by SAB Company (Pearland, TX, USA). GAPDH or β-actin rabbit polyclonal antibody (Proteintech, Los Angeles, CA, USA) was used as internal control. Horseradish peroxidase (HRP)-conjugated goat anti-mouse IgG or anti-rabbit IgG (Proteintech) was used as secondary antibody for enhanced chemiluminescence (ECL) detection in western blot. The Annexin V-FITC/PI double immunofluorescence staining kit was purchased from Beyotime Company (Shanghai, China).

### 2.3. Cytotoxicity Assay of Baicalin

Baicalin was serially diluted in a medium containing 2% FBS with the concentrations of 0, 6.25, 12.5, 25, 50, 100, 200, and 400 μg/mL, respectively. An aliquot of baicalin solution (100 μL) was added into a 96-well plate with monolayer cells, and each experiment was repeated in triplicate. The cells were cultured for 48 h at 37 °C with 5% CO_2_ and then tested for cell survival using MTT (3-[4.5-dimethylthiazol-2-yl]-2,5-diphenyl tetrazolium bromide; Sigma, Saint Louis, MO, USA) assay (Beyotime, Shanghai, China). The cytopathic concentration 50% (CC_50_) of baicalin was calculated using probit progression [20,21].

### 2.4. Plaque Reduction Assay

Virus infection analysis was performed by plaque reduction assay as described previously [22] with some modifications. Briefly, a total of RD cells (3 × 10^5^) were seeded into six-well plates and cultured until 80% confluency. After viral absorption for one hour at 4 °C, the culture supernatants were replaced with pre-warmed fresh DMEM containing 2% FBS and 1.5% methyl cellulose for five days’ continuous culture at 37 °C with 5% CO_2_. The cells were then fixed with 4% paraformaldehyde for 4 h, stained with 5% crystal violet staining for 15 min, and washed with running water. Plaque formation count was calculated under an invert microscope.

### 2.5. Antiviral Activity of Baicalin

To investigate the anti-EV71 activity of the baicalin, the experiments were performed according to the previously described method [22]. Baicalin was diluted in serum-free DMEM to 0, 6.25, 12.5, 25, and 50 μg/mL. Then three independent assays were performed to analyze the characteristics of baicalin against EV71. After virus absorption for 1 h, the medium was aspirated from the well to remove the unabsorbed virus, and cells were washed three times with serum-free DMEM, then the virus-infected RD cells were treated with different concentrations of baicalin for the antiviral effect test. EV71 was pre-treated with different concentrations of baicalin for 4 h at 37 °C with 5% CO_2_; subsequently, the culture supernatants were replaced with different concentrations of baicalin after 1 h of absorption of treated-EV71 for the direct virucidal effect test. RD cells were pre-incubated with different concentrations of baicalin for 4 h at 37 °C with 5% CO_2,_ and washed three times with serum-free DMEM, then infected with EV71 (MOI = 5) for 1 h for virus absorption. The culture supernatants were replaced with fresh DMEM containing 2% FBS continuous culture at 37 °C with 5% CO_2_ for the prophylactic effect test. Finally, EV71-infected cells and culture supernatants were collected at 48 h post infection (p.i.) for plaque reduction assay. The number of plaque formation was counted under an inverted microscope, and the percentage of inhibition was calculated as follows: [(mean number of plaques in control) − (mean number of plaques in sample)]/(mean number of plaques in control) × 100; each assay was repeated three times [23].

### 2.6. Virus Absorption Assay and Time Course Analysis

To understand the correlation between baicalin and virus absorption [24], 50 µg/mL of baicalin was added into monolayer RD cells with EV71 at the same time or 1 h after EV71 absorption, and then the infected cells and culture supernatants were collected at 0 h, 4 h, 8 h, 16 h, 24 h, and 36 h p.i. for plaque reduction assay. Time course analysis was performed according to the previously described method [25]. RD cells (3 × 10^5^) grown in 12-well plate were infected with EV71 (MOI = 5) at 4 °C for 1 h for viral absorption. Baicalin (50 μg/mL) was added into RD cells at 0 h, 2 h, 4 h, 6 h, 8 h, 10 h, 12 h, 16 h, and 24 h p.i., respectively. The supernatants of cell lysates were collected at 25 h p.i. for viral plaque reduction assay.

### 2.7. Reverse Transcription PCR (RT-PCR)

Total RNA was extracted from the infected cells and culture supernatants by the Trizol method. The primers of EV71/VP1, 2A, 3C, and 3D were designed and synthesized by Sangon Biotech Company (Shanghai, China) and shown in Table 1. Real-time PCR was done using the Omega One step RT-PCR kit (Omega, Norcross, GA, USA) under the following conditions: 5 min at 94 °C, followed by 40 cycles of 30 s at 94 °C, 30 s at 50 °C, and 60 s at 72 °C. The amplification products were detected by electrophoresis with 2% agarose gels.

### 2.8. Western Blot Analysis

Total protein was extracted and the concentration was determined by BCA assay. Twenty microliters of the sample was loaded into each well for sodium dodecyl sulfate polyacrylamide gel electrophoresis (SDS-PAGE) and then transferred to a PVDF membrane (Millipore, Billerica, MA, USA). The membrane was blocked for 2 h with 5% nonfat dry milk solution in TBST (Tris-buffered saline containing 0.1% Tween-20) at room temperature and incubated with primary antibodies (VP1, 1:500; 2A, 1:1000; 3C, 1:1000; and 3D, 1:500) overnight at 4 °C, and followed by incubation with secondary antibody conjugated with horseradish peroxidase (Proteintech) for 1 h. The immunoreactive bands were detected by Super RX film (Fujifilm, Tokyo, Japan) and quantified by ImageQuant densitometric analysis (Molecular Dynamics, Sunnyvale, CA, USA).

**Table 1:** The primers of EV71/VP1, 2A, 3C, and 3D genes.

Genes	Primer sequence	Length of product (bp)
VP1	FP: 5'-TGGCAGATGTGATTGAGAG-3'	137
	RP: 5'-GGCTTGAAGTGCTGGTA-3'	
2A	FP: 5'-ACCTTAGGGTAGTAAACAGACAC-3'	262
	RP: 5'-GCAAGCATAAGATGGGACT-3'	
3C	FP: 5'-GACCATCTGGGTAGAGCAT-3'	143
	RP: 5'-CTTCTGGGATAAACTTGGTG-3'	
3D	FP: 5'-GGAAGTCTCGCCTGATTG-3'	478
	RP: 5'-CAGCAGGAGTCATAGTCAGC-3'	
GAPDH	FP: 5'-GGATTTGGTCGTATTGGG-3'	205
	RP: 5'-GGAAGATGGTGATGGGATT-3'	

FP: Forward primer; RP: Reverse primer

### 2.9. RD Cell Apoptosis by Annexin V-FITC/PI Dual Staining and Flow Cytometry

The cells were divided into a control group, a baicalin treatment group (50 μg/mL of baicalin was added into RD cells), an EV71 + baicalin treatment group (50 μg/mL of baicalin was added into RD cells at 1 h after EV71 absorption), and an EV71 infection group. EDTA-free trypsin was used to digest cells into a single-cell suspension, which was slowly dropped onto the cover slips pre-placed in six-well plates for continuous culture at 37 °C with CO_2_. When 80% confluency was reached, the cells were infected with EV71 (MOI = 5) and continuous culture for 20 h p.i. Subsequently, the cover slips were slowly washed with pre-warmed PBS (37 °C) three times (5 min each) to eliminate residual proteins. Annexin V-FITC/PI dual staining was immediately carried out, and stained cells were observed under an inverted fluorescence microscope. After 30 s of EDTA-free trypsin digestion, 4 × 10^5^ cells were collected for annexin V-FITC/PI dual staining and then analyzed by flow cytometry within 1 h.

### 2.10. Statistical Analyses

All data were presented as mean ± SD. Statistical difference was determined by ANOVA or Student’s *t*-test as indicated in figures and legends. A significant difference was determined to be when the *p*-value was less than 0.05.

## 3. Results

### 3.1. Cytotoxicity of Baicalin on RD Cells

**Figure 1:**
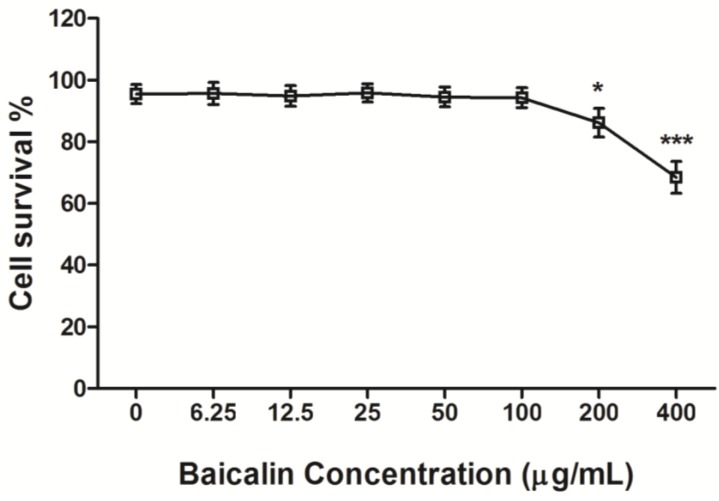
The cytotoxicity of baicalin on RD cell viability. Baicalin was serially diluted in a medium containing 2% FBS with concentrations of 0, 6.25, 12.5, 25, 50, 100, 200, and 400 μg/mL, respectively. Subsequently, the cytotoxicity of baicalin on RD cells was determined by MTT assay. The absorbance was measured using a microplate reader at 570 nm. The CC_50_ value was calculated as the extract concentration necessary to reduce cell viability by 50%. Data were expressed as mean ± SD from three independent experiments and analyzed by one-way ANOVA (* *p* < 0.05, *** *p* < 0.001).

To understand the cytotoxicity of baicalin on RD cells, MTT assay was used to determine the cell viability at 48 h after baicalin treatment with different concentrations. We found that baicalin (≤100 μg/mL) failed to distinctly affect cell viability, and its CC_50_ was 823.53 μg/mL. This indicated that baicalin had only weak cytotoxicity on RD cells (Figure 1).

### 3.2. The Antiviral Efficiency of Baicalin on EV71 Replication and Absorption

**Figure 2:**
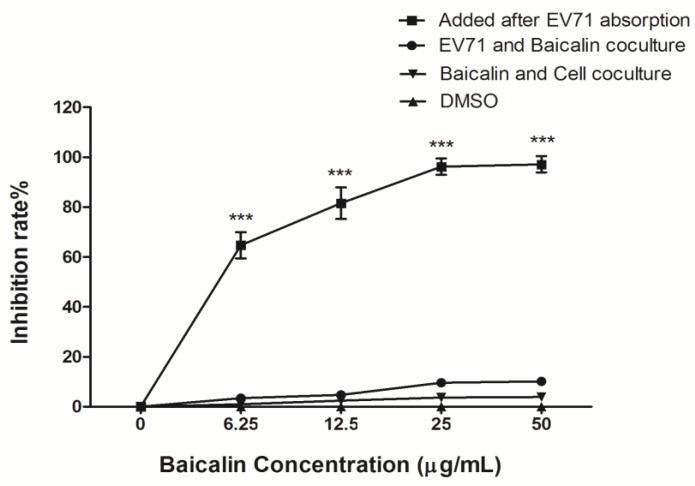
The inhibitory effect of baicalin on EV71 replication. Baicalin was diluted in serum-free DMEM to 0, 6.25, 12.5, 25, and 50 μg/mL. Baicalin-free was considered as the EV71 infection control group. The virus-infected RD cells were treated with different concentrations of baicalin after 1 h of virus absorption to detect the inhibiting virus effect of baicalin. EV71 was pre-treated with different concentrations of baicalin to detect the directly virucidal effect of baicalin. RD cells were pre-incubated with different concentrations of baicalin to detect the prophylactic effect of baicalin. The infected cells and culture supernatants were collected at 48 h p.i. for plaque reduction assay. The data were expressed as mean ± SD from three independent experiments and analyzed by two-way ANOVA with Bonferroni *post hoc* tests (*** *p* < 0.001).

**Figure 3:**
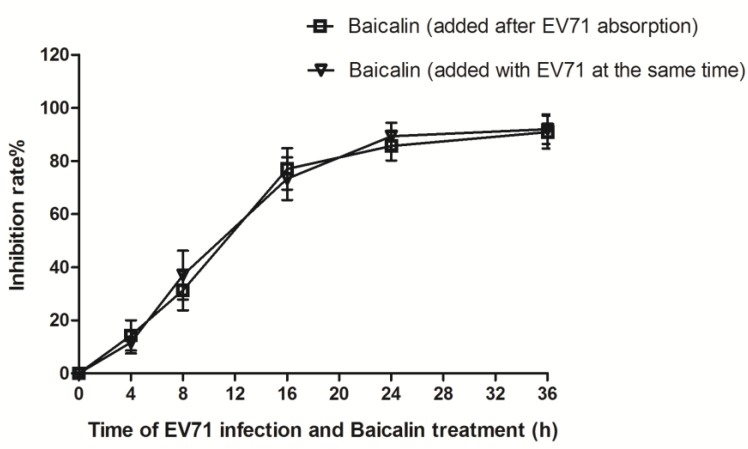
The effect of baicalin on EV71 absorption. Fifty micrograms per milliliter of baicalin were added into monolayer RD cells with EV71 at the same time or 1 h after EV71 absorption. The infected cells and supernatants were collected at 0 h, 4 h, 8 h, 16 h, 24 h, and 36 h p.i. for plaque reduction assay. Data were expressed as mean ± SD from three independent experiments and analyzed by one-way ANOVA.

The changes of viral titer were detected by plaque reduction assay in cell supernatants at 48 h p.i. One hour after virus absorption, RD cells were treated with baicalin (6.25, 12.5, 25, and 50 μg/mL), which showed that the inhibitory rates were 64.7 ± 5.4%, 81.5 ± 6.4%, 96.2 ± 3.8%, and 97.1 ± 2.8%, respectively. The 50% inhibitory concentration (IC_50_) of baicalin on EV71 replication was 4.96 μg/mL. When EV71 was pre-treated with serially diluted baicalin for 4 h and then infected RD cells, it showed that the inhibitory rates were 3.5 ± 2.4%, 4.8 ± 1.7%, 9.6 ± 3.1%, and 10.2 ± 2.8%, respectively. When RD cells were pre-incubated with the same concentrations of baicalin for 4 h and then EV71 infected RD cells, the inhibition rates were 1.0 ± 0.9%, 2.5 ± 0.7%, 3.7 ± 0.7%, and 3.9 ± 1.4%, respectively. Mock treatment with DMSO did not result in an inhibitory effect on RD cells (Figure 2). In addition, the inhibition rates of baicalin against EV71 showed no significant differences in virus absorption assay (Figure 3). These results indicated that baicalin exhibited a strong antiviral activity on EV71 in a dose-dependent manner, but any direct virucidal or prophylactic activities and inhibitory viral absorption were not observed.

### 3.3. Time-Course Analysis of Baicalin on EV71 Replication

**Figure 4:**
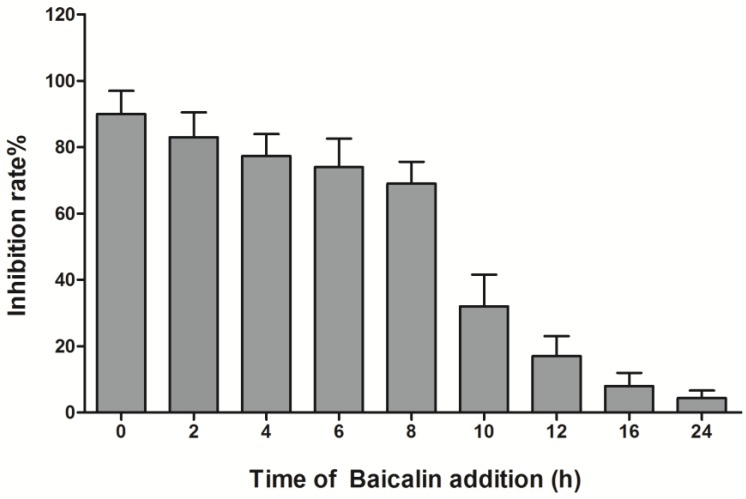
The inhibitory activity of baicalin on EV71 replication. RD cells (5 × 10^6^) were infected with EV71 (MOI = 5) , and then baicalin (50 μg/mL) was added at 0 h, 2 h, 4 h, 6 h, 8 h, 10 h, 12 h, 16 h, and 24 h p.i., respectively. The infected cells and supernatants were collected at 25 h p.i., and virus titers were measured by plaque reduction assay. Data were expressed as mean ± SD from three independent experiments and analyzed by one-way ANOVA.

To analyze the antiviral effect of baicalin at what point in the viral replication cycle of EV71, baicalin (50 μg/mL) was added to RD cells at 0 h, 2 h, 4 h, 6 h, 8 h, 10 h, 12 h, 16 h, and 24 h p.i., respectively. Time-course analysis showed that baicalin could remarkably suppress EV71 replication in RD cells at 0–8 h p.i., while only partial or minimal inhibitory effect was observed at 10–24 h p.i. Thus, it is postulated that the effect of baicalin on EV71 mainly occurs in the early stage of virus infection (Figure 4).

### 3.4. Effects of Baicalin on the Expression of EV71/VP1, 2A, 3C, and 3D

**Figure 5:**
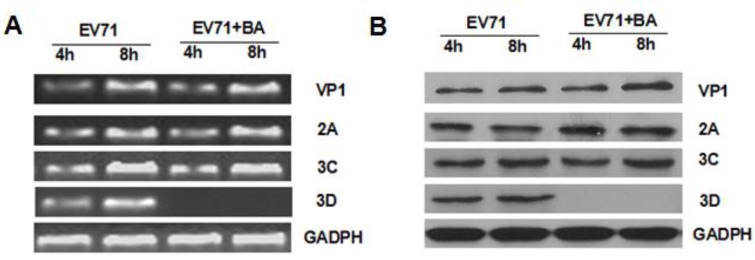
The effects of baicalin on EV71 mRNA and protein. EV71:EV71 infection; EV71 + BA:EV71 infected-RD cells treated with baicalin (50 μg/mL). (**A**) 3×10^5^ of RD cells were infected with EV71 (MOI = 5) for 1 h and then treated with baicalin or maintenance medium. Total RNA was extracted at 8 h and 24 h p.i. and detected by agarose gel electrophoresis; (**B**) The total protein of EV71-infected cells was extracted at 8 h and 24 h p.i. and detected by Western blot.

To analyze the impact of baicalin on EV71/VP1, 2A, 3C, and 3D transcription and translation, the total RNA was extracted from EV71-infected cells treated with or without baicalin (50 μg/mL) at 8 h and 24 h p.i. Subsequently, RT-PCR products were detected by agarose gel electrophoresis. The mRNA expressions of VP1, 2A, 3C, and 3D significantly increased at 8 h and 24 h p.i. Pretreated with baicalin (50 μg/mL), the mRNA level of 3D was surprisingly inhibited, but that of VP1, 2A, and 3C was not affected at all (Figure 5A). Consistent with mRNA expression, baicalin inhibited the expression of 3D polymerase at 8 h and 24 h p.i. (Figure 5B).

### 3.5. The Effect of Baicalin on EV71-Induced Apoptosis in RD Cells

**Figure 6:**
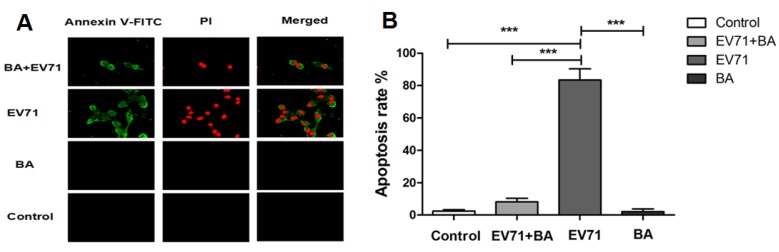

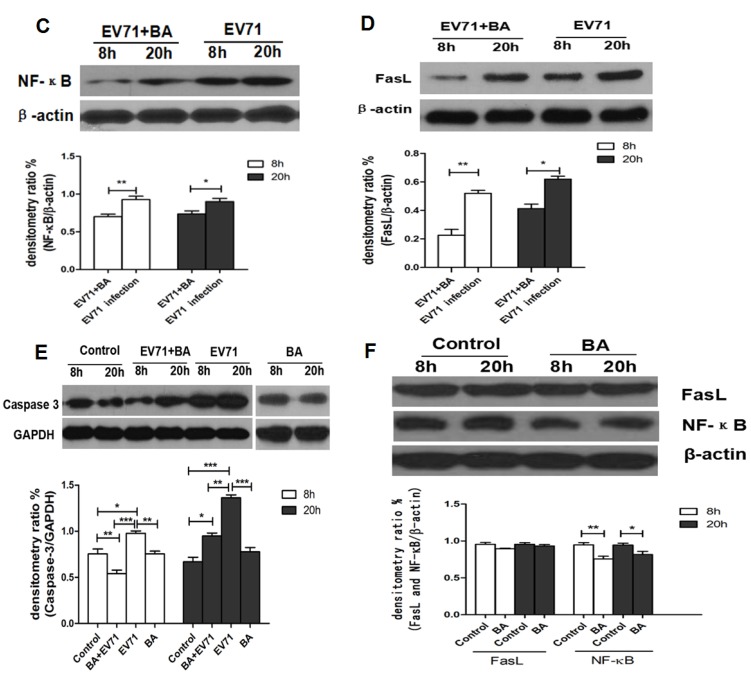
The inhibitory effect of baicalin on EV71-induced apoptosis. Control: Uninfected RD cells; BA: RD treated with baicalin (50 μg/mL); EV71+BA: EV71-infected RD treated with baicalin (50 μg/mL); EV71: EV71-infected RD cells. The apoptosis of EV71-infected RD cells was observed under fluorescence microscopy (**A**) and detected by flow cytometry (**B**) at 20 h p.i. Western blot analysis for FasL, caspase-3, and NF-κB in EV71-infected RD cells at 8 h and 20 h p.i. (**C**–**F**). ImageJ software was used to quantify protein bands. All data were presented as mean ± SD of three independent experiments and analyzed by one-way ANOVA followed by a Dunnett’s Multiple Comparison Test (* *p* < 0.05, ** *p* < 0.01, *** *p* < 0.001).

EV71 infection often induces apoptosis in various host cells. To understand the effect of baicalin on EV71-infected RD cells, the cell viability was observed under a fluorescence microscopy and detected by flow cytometry. Compared with the control group, baicalin could effectively inhibit the apoptosis of EV71-infected RD cells (Figure 6A,B). EV71 infection remarkably enhanced the expressions of FasL and caspase-3 at 20 h p.i., and further stimulated the activation of Fas/FasL-mediated apoptosis pathway. Compared with the EV71 infection group, the expression of FasL was inhibited in EV71-infected RD cells by baicalin treatment at 8 h and 20 h p.i. (Figure 6C). Similarly, the expression of caspase-3 was also suppressed significantly in EV71-infected RD cell by baicalin treatment at 8h and 20 h p.i. (Figure 6D). In addition, the expression levels of NF-κB p65 were elevated at 8h and 20 h p.i., whereas baicalin could decrease the expression of NF-κB p65, which might inhibit the production of proinflammatory cytokines and mitigate RD cell damage [26,27] (Figure 6E,F).

## 4. Discussion

EV71 is a virus with the advantages of genetic diversity and rapid evolution. Currently, the effect of antiviral drugs, vaccines, or interferons on EV71 infection is limited. Flavonoids are widely distributed natural products with broad biological activities. Moreover, several naturally occurring dietary flavonoids could exhibit antiviral effects on EV71. For example, apigenin inhibits EV71 infection through either disrupting viral RNA association with hnRNP A1 and A2 proteins or suppressing viral internal ribosome entry site (IRES) activity and modulating cellular JNK pathway [28]. Luteolin targets the post-attachment stage of EV71 and CA16 infection by inhibiting viral RNA replication [29], whereas 7-hydroxyisoflavone acts at an early step of EV71 replication [29]. Baicalin is a flavonoid compound purified from the root of *Scutellaria*, which has been reported to possess a variety of biological activities. For example, baicalin can form complexes with many types of chemokines to block their binding to corresponding receptors, which significantly reduces the chemokine-induced invasion of neutrophils and inhibits the activity of cyclooxygenase-2 [30]. Meanwhile, baicalin not only inhibits the HBV core promoter gene blocking HBV production [31], but also resists influenza virus H1N1infection through inhibiting NA activity and regulating viral NS1 protein function or cytokine production [32]. To date, it is still poorly understood whether baicalin can inhibit EV71 infection. Here, we found that baicalin (≤100 μg/mL) did not have any cytotoxicity on RD cell viability, and its CC_50_ was 823.53 μg/mL. Meanwhile, baicalin exhibited a strong antiviral effect on EV71 in a dose-dependent manner, while its IC_50_ on EV71 was only 4.96 μg/mL, indicating that baicalin possesses the characteristics of high efficiency and low toxicity. In fact, viral inactivation is not equal to death, which maintains the basic virus antigenicity but not the infectivity. As previously reported, some extracts of plant or algae species exhibit antiviral activity against HSV, DENV, or HSV-1 by direct virus inactivation [33,34,35]. However, no direct virucidal effect or prophylactic effect were observed for baicalin against EV71 infection. Compared with baicalin addition before or after EV71 absorption, titers of EV71 in supernatants were similar, indicating that baicalin might be independent of inhibiting viral absorption. In addition, baicalin significantly suppressed EV71 replication in RD cells at 0–8 h p.i., while only partial or minimal inhibitory effect was observed at 10–24 h p.i. This indicates that the antiviral effects of baicalin occur mainly at the early stages of EV71 infection.

EV71 has a single-stranded positive-sense RNA encoding four capsid proteins (VP1, VP2, VP3, and VP4) and seven nonstructural proteins (2A, 2B, 2C, 3A, VPg, 3C, and 3D) [36] that are involved in the life cycle of viruses including absorption, invasion, shelling, and replication. The capsid proteins VP1, VP2, and VP3 are antigenic and recognize the receptors on the surface of specific host cells. The nonstructural genes play important roles in virus replication [37]. In particular, 2A, 3C, and 3D proteins perform multiple enzymatic functions. For example, 2A protease catalyzes the cleavage of the peptide bond of precursor proteins, P1 and P2, to complete the primary processing of polypeptides, while 3C protease or 3CD precursor is involved in the secondary processing of polypeptides [38,39]. In addition, 3D polymerase, a viral RNA-dependent RNA polymerase that is not found in host cells, is critical in the synthesis of viral plus and minus strands, participates in VPg uridylation, and initiates RNA replication [40]. Furthermore, these viral proteases have become the preferred targets for the current development of antiviral drugs [41]. As previously reported, aurintricarboxylic acid (ATA) was able to inhibit the 3D RNA-dependent RNA polymerase (RdRp) activity of EV71, while neither the IRES-mediated translation of viral polyprotein nor the viral 2A and 3C protease activity was affected [29]. In our study, we also found that the expression of EV71/3D was significantly blocked by BA treatment at early stages of EV71 infection, while 2A and 3C protease were not affected. In addition, EV71 is a positive-sense single-stranded RNA virus. On the one hand, its genome is directly utilized as if it were mRNA, with host ribosomes translating it into a large polyprotein precursor, *i.e.*, it is proteolytically cleaved by viral 2A and 3C proteases to form various structural and non-structural viral proteins [42,43]. On the other hand, one of the non-structural viral proteins is RNA-dependent RNA polymerase (a major component of the viral replication complex), which initiated *de novo* transcription with a poly(C) template and genome RNA to form a double-stranded replicative form [40]. In turn, this directs the formation of new virions. It is important to note that baicalin may not solely rely on EV71/3D inhibition to exert its effects on EV71 replication. Therefore, further investigations are needed to explore whether baicalin also affects other viral or cellular functions.

Apoptosis is an important mechanism for the maintenance of homeostasis in mammals, and it also plays an important role in preventing virus replication, dissemination, or persistent infection [44,45,46]. Fas receptor, an important cell surface receptor protein of the TNF receptor family, is also known as CD95. Once Fas binds with FasL, they stimulate the activation of caspase-8 and caspase-3 and induce apoptosis [45]. Virus infection often leads to apoptosis of host cells. For example, rotavirus resists host cell antiviral response by inhibiting STAT1, STAT2, and NF-κB signaling pathways to facilitate its own replication [47], while dengue virus subgenomic RNA induces apoptosis by regulating the Bcl-2-mediated PI3K/Akt signaling pathway [48]. In addition, the transcription factor NF-κB is considered one of the key regulators of inflammatory cytokine secretions, cell proliferation, transformation, and tumor progression [49]. Some viruses, such as EV71, influenza A virus, and HSV-1, require the activation of the NF-κB signaling pathway for efficient viral replication [50,51,52]. In our previous study, we discovered that FasL/Fas and NF-κB/RelA signaling pathways were significantly activated with EV71-induced apoptosis in RD cells [8]. Here, we showed that EV71 infection enhanced the expressions of FasL and caspase-3 in RD cells, which could be significantly inhibited by baicalin. Meanwhile, EV71 infection stimulates the expression of NF-κB, which can be suppressed by baicalin treatment. Therefore, we propose that baicalin could inhibit the apoptosis of EV71-infected RD cells by way of the Fas/FasL signaling pathway, as well as decrease the secretion of cytokines by downregulating NF-κB signaling pathways.

## 5. Conclusions

In summary, baicalin exhibited a strong antiviral effect on EV71 replication at early stages of infection with high efficiency and low toxicity. The antiviral effects are probably correlated with the blockage of EV71/3D mRNA and polymerase. In addition, baicalin may inhibit the apoptosis of EV71-infected RD cells through activating Fas/FasL signaling pathways and blocking NF-κB signaling pathways. Therefore, baicalin can significantly inhibit EV71 infection and become a potential drug for the treatment of EV71 infection. However, the inhibition efficiency of baicalin on EV71 infection should be further evaluated by plasmid transfection experiment, as well as the 3D functions through polymerase elongation assay and animal models, in order to finally use it for the treatment of viral infection.
